# Brain perfusion CT compared with^15^O-H_2_O-PET in healthy subjects

**DOI:** 10.1186/2191-219X-1-28

**Published:** 2011-11-18

**Authors:** Julie Marie Grüner, Rune Paamand, Liselotte Højgaard, Ian Law

**Affiliations:** 1 Department of Clinical Physiology, Nuclear Medicine and PET, Rigshospitalet, University of Copenhagen, Blegdamsvej 9, Copenhagen, 2100, Denmark

**Keywords:** brain perfusion imaging, PET, oxygen radioisotopes, perfusion CT, healthy human subjects

## Abstract

**Background:**

Regional cerebral blood flow [rCBF] measurements are valuable for identifying angiogenically active tumours, and perfusion computed tomography [CT] has been suggested for that purpose. This study aimed to validate rCBF measurements by perfusion CT with positron-emission tomography [PET] and^15^O-labelled water [^15^O-H_2_O] in healthy subjects.

**Methods:**

RCBF was measured twice in 12 healthy subjects with^15^O-H_2_O PET and once with perfusion CT performed over the basal ganglia. Matching rCBF values in regions of interest were compared.

**Results:**

Measured with perfusion CT, rCBF was significantly and systematically overestimated. White matter rCBF values were 17.4 ± 2.0 (mean ± SD) mL min^-1 ^100 g^-1 ^for PET and 21.8 ± 3.4 mL min^-1 ^100 g^-1 ^for perfusion CT. Grey matter rCBF values were 48.7 ± 5.0 mL min^-1 ^100 g^-1 ^for PET and 71.8 ± 8.0 mL min^-1 ^100 g^-1 ^for perfusion CT. The overestimation of grey matter rCBF could be reduced from 47% to 20% after normalization to white matter rCBF, but the difference was still significant.

**Conclusion:**

RCBF measured with perfusion CT does contain perfusion information, but neither quantitative nor relative values can substitute rCBF measured by^15^O-H_2_O PET yet. This, however, does not necessarily preclude a useful role in patient management.

## Background

Quantitative measurement of the regional cerebral blood flow [rCBF] is a fundamental physiological parameter for characterizing the status of the brain tissue. RCBF measurements have important clinical implications in defining tissue ischemia [1,2], in diagnosing neurodegenerative diseases [3], and in locating and monitoring angiogenically active tumour tissues [4,5]. Positron-emission tomography [PET] measurements using a freely diffusible tissue tracer, oxygen-15-labelled water [^15^O-H_2_O], is regarded as one of the rCBF gold standards [6-8]. This cumbersome technique requires on-line tracer production from a cyclotron and continuous arterial blood sampling. It is expensive, technically demanding, and traumatic for the patient, and is rarely found outside specialized hospital units. An attractive alternative to PET would be dynamic contrast-enhanced computed tomography [CT] or perfusion CT. Perfusion CT methodology has improved profoundly in recent years. It is now a practical technique for the clinical environment due to the development and broad introduction of fast multidetector row CT systems, increasing computer power, new image acquisition protocols, and commercially available perfusion softwares [1]. It is also relatively cheap, easy, and rapid to perform. Furthermore, in many acute medical and surgical conditions, such as stroke, head injury, and subarachnoid haemorrhage, and in radiotherapy planning, cerebral CT scanning is often the primary imaging modality of choice. This makes perfusion CT particularly applicable for additional tissue characterization [9]. Another interesting application has arisen with the advent of hybrid imaging techniques such as PET/CT and single photon emission computed tomography/CT. In the same examination, and with only minor additional scan time, perfusion CT can be considered as an adjunct to further visualize the vascular physiology of relevant lesions. This complements and extends the physiological tissue information obtained by PET, e.g. the glucose metabolism using^18^F-fluorodeoxyglucose [10] or hypoxia using^18^F-fluoromisonidazole [11].

However, the underlying kinetic models for rCBF measured by perfusion CT and PET with^15^O-H_2_O differ fundamentally. Perfusion CT is based on the dynamic behaviour of an intravascular contrast agent, while^15^O-H_2_O PET models a freely diffusible tissue tracer. To investigate this further, we compared the rCBF measurements of the two techniques directly in the same healthy subjects on the same day within a few hours.

## Materials and methods

### Subjects

Seventeen healthy volunteers were recruited to the protocol and scanned from August 2008 to April 2009. All subjects underwent a structured interview of health history. The exclusion criteria were a known neurological disease, migraine, reduced renal function, pregnancy, and a known allergy against iodinated contrast media. All subjects gave oral and written informed consents according to the Helsinki II Declaration. The plasma creatinine levels of all subjects were measured within 2 weeks before the scan and were all within a normal range. One subject was excluded because of a technical failure in the CT iv-contrast power injector; two subjects were excluded because of failure in the arterial blood sampling during PET scanning; one subject was excluded as the arterial catheter could not be placed correctly; and in one subject, the rCBF PET kinetic modelling failed. Consequently, 12 volunteers (nine females, three males) were available for analysis with a median age of 24 years (range 20 to 26 years). The protocol was approved by the Committee on Biomedical Research for the Capital Region of Denmark (protocol number H-A-2008-055).

### PET protocol

#### Scanner

A dedicated brain high-resolution research tomograph PET scanner (CTI/Siemens, Knoxville, TN, USA) was used for all PET scans. This scanner has an axial field of view of 25 cm and a near-isotropic resolution of 2 mm.

#### PET tracer

An 800-MBq^15^O-H_2_O was produced on-line and injected intravenously in an antecubital vein via an automatic water injection system [AWIS] (1997, Scansys, Værløse, Denmark). AWIS delivered a 16-mL bolus over 10 s with both pre- and after-flush of an inert saline solution [7]. Each volunteer had two tracer injections. A short indwelling catheter was placed in the non-dominant radial artery under local anaesthesia for blood sampling.

#### Image acquisition

During scanning, the subject's head was rested in a foam-cushioned headrest, and a head strap was used to minimize head movement. Initially, a 6-min transmission scan with a rotating^137^Cs single-photon point source was performed for attenuation correction. The 7-min emission scans were acquired in a three-dimensional [3D] list mode and initiated immediately before tracer injection. The interscan interval was at least 10 min to allow for isotope decay.

For kinetic modelling, arterial blood was sampled continuously during the scans using an automatic blood sampling system [ABSS] (Allogg, Mariefred, Sweden) set to draw arterial blood at a constant speed of 8 mL/min with its activity measured every 0.5 s. The inner diameter of the tube connected to the arterial catheter was 1.0 mm. Immediately after the scan, 2 mL of arterial blood was drawn for blood gas analysis to evaluate the physiologic respiratory state of the subjects. The samples were analysed for arterial partial pressures of oxygen [P_a_O_2_] and carbon dioxide [P_a_CO_2_], saturation level of oxygen [sO_2_], and haemoglobin concentration [ctHb] (ABL 700 Series, Radiometer Medical, Copenhagen, Denmark). The detectors in the ABSS and the PET scanner were cross-calibrated against an independent dose calibrator so that all data could be reported in radioactivity concentration (in becquerel per millilitre).

#### Image reconstruction

Dynamic images were reconstructed using a 3D-ordered subset expectation maximization algorithm with correction for the measured point spread function into 40 frames per scan with durations of 1 × 30, 18 × 5, 9 × 10, 10 × 15, and 2 × 30 s. Each frame consisted of 207 image planes in a 256 × 256 matrix with an isotropic voxel size of 1.22 × 1.22 × 1.22 mm^3^. The first 30-s frame was designed to accommodate the tracer delay from injection to the brain tissue. All images were corrected for dead time and scatter and filtered with a 3D Gaussian 5-mm filter.

#### PET CBF calculation

Using a commercially available software package, PMOD 3.0 (PMOD Technologies, Zürich, Switzerland), the dynamic images of the first 210 s following the arrival of activity to the brain and the delay- and dispersion-corrected arterial input functions [12] were fitted by Alpert's one-tissue compartment model [13] according to Equation 1:

(1) $$C_{\text{t}}\left(t\right)=fC_{\text{a}}\left(t\right)⊗e^{-\left(f∕\text{Vd}\right)t}$$

where *C*_t_(*t*) denotes tissue activity concentration (in becquerel per millilitre), *C*_a_(*t*) is the measured arterial input function (in becquerel per millilitre), *f *is the rCBF (in millilitre per minute per 100 g), and Vd (in millilitre per gram) is the fitted volume of distribution. ⊗ represents the convolution operation. This generated the parametric images of rCBF.

#### Radiation dose

The dose equivalent following PET transmission and emission scans was in a total of 1.6 mSv: 0.1 mSv for the transmission scan and 0.74 mSv for each emission scan [14].

### CT protocol

#### Scanner

A Biograph 40 TruePoint PET/CT scanner (Siemens, Knoxville, TN, USA) was used.

#### Contrast media

A preheated iso-osmolar iodine contrast medium OptiRay 350 (Ioversol 350 mg/mL, Tyco Healthcare, Neustadt an der Donau, Bayern, Germany) was injected intravenously by a power injector (OptiVantage DH Injection System, Liebel-Flarsheim, Cincinnati, OH, USA) as a short bolus of 40 mL (8 mL/s) through a catheter in the antecubital vein followed by 20 mL of saline solution.

#### Image acquisition

In nine subjects, PET scanning was performed before CT, and in three subjects, CT before PET. A lateral scout scan at the angle of the meato-orbital plane was performed followed by a non-enhanced low dose CT (120 kVp, 40 mAs). This guided the selection of four contiguous transaxial slices at the anatomical level of the third ventricle and through the basal ganglia, which is the level most frequently used in perfusion CT stroke evaluations. The orbits were not in the field of view to avoid unnecessary irradiation to the lens. The contrast medium was injected 4 s before the initiation of the dynamic scan. The dynamic scan consisted of 160 images: one image for 40 s over four slices of 7.2-mm thick at 80 kVp and 120 mAs. The arterial cannula from the PET scan was kept in place and used to draw arterial blood for blood gas analysis immediately after the scan.

#### Image reconstruction and rCBF calculation

Each slice was reconstructed into a 512 × 512 image matrix using a H30s medium smooth kernel. Voxel dimensions were non-isotropic 0.44 × 0.44 × 7.2 mm^3^. The rCBF was calculated in a semi-automated manner using a commercial software, Syngo Neuro Perfusion CT 2006A (Siemens, Knoxville, TN, USA). After segmentation and removal of an extra cerebral tissue, a circular reference region of interest [ROI] was defined automatically in the occipital part of the superior sagittal sinus. Maximum intensity projection [MIP] CTs were reconstructed to enhance areas of high radiodensity that are useful for identifying vascular structures. Regions with larger vessels were excluded in the rCBF assessments by thresholding the MIP CT image by 15% of the maximal value corresponding to a regional cerebral blood volume [rCBV] threshold of 14.4 mL/100 mL. The arterial input function was derived from the time-attenuation curve from ROIs comprising both anterior cerebral arteries in cross section, and the rCBF (in millilitre per minute per 100 mL) was calculated using a deconvolution approach. By dividing with the brain tissue density of 1.04 g/mL, the rCBF values were converted into units per weight tissue as for rCBF PET.

#### Radiation dose

The effective dose equivalent was 2.9 mSv for the CT perfusion protocol.

## Data analysis

### Image co-registration and ROIs

**Figure 1:**
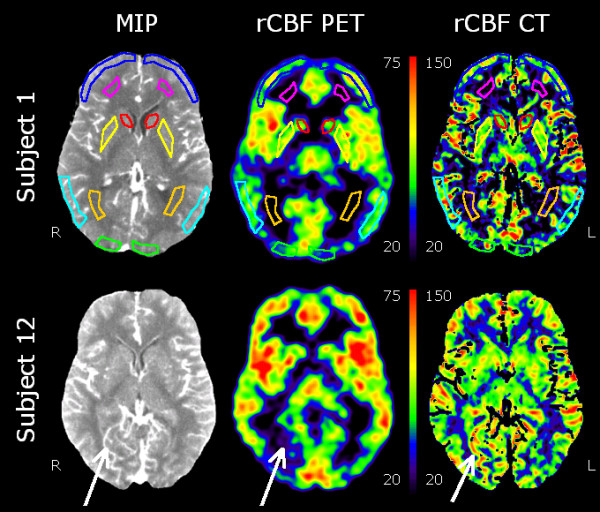
**Co-registered transaxial slices through the level of the basal ganglia**. Co-registered transaxial slices showing MIP from perfusion CT integrated over 40 s (left), the rCBF images using PET (centre), and perfusion CT (right) quantified in millilitre per minute per 100 g. For a better comparison, the rCBF PET and rCBF CT images are displayed in different scales. The top row shows the location and configuration of ROIs in the frontal cortex (dark blue), parietal cortex (light blue), occipital cortex (green), caudate nucleus (red), putamen (yellow), frontal white matter (purple), and occipital white matter (orange). High-intensity areas in the MIP images represent larger vascular volumes around the vessels of the cortex and insula, the choroid plexus, and the sinuses. These are partly, but not completely, removed by masking the rCBF CT images. This discrepancy between the two techniques is evident around a right-sided occipital vein that is visualised in rCBF CT, but not in rCBF PET (white arrow, bottom row).

Using PMOD, rCBF PET, rCBF CT, and MIP CT images were co-registered to the low-dose CT scan of the head to a final voxel size of 0.5 × 0.5 × 1.5 mm^3^. This was done to insure that all ROIs referred to and included an identical tissue composition across techniques. On the MIP CT images, 14 ROIs were drawn in symmetrical areas over the grey matter in the head of the caudate nuclei, putamen, frontal cortex, temporal/parietal cortex, occipital cortex, and anterior and posterior white matter (Figure 1, top). By using a masking technique on the rCBF CT images, values below zero inside ROIs were excluded from analysis. In one subject, the putamen could not be drawn because it was only partly included in the scanned area. Finally, the 14 ROIs were projected onto identical areas of the masked parametric rCBF PET and rCBF CT images for quantification. The tissue volume in the ROIs ranged from 109 to 407 mm^3 ^(average 247 mm^3^). We then calculated the volume-weighted average white and grey matter values based on the selected ROIs.

### Statistical methods

The statistical analysis was conducted using MATLAB (MathWorks Inc., Natick, MA, USA). Paired *t *tests with a two-tailed significance level of *α *= 0.05 was used to evaluate the blood gas data. A similar method was used for the analyses between rCBF CT and the average of two rCBF PET scans for white and grey matter ROIs.

In the quantitative assessment of rCBF using PET, CT, and magnetic resonance imaging [MRI], it is recognized that the global variation in CBF may have a significant impact on the regional variation [15-18]. We, thus, repeated the comparison of grey matter values after normalization to the volume-weighted average of the four white matter ROIs. The two-tailed significance level for the paired *t *tests was at *α *= 0.05, but a Bonferroni correction was applied for multiple non-independent comparisons. Thus, the thresholds were *p *< 0.0036 (14 comparisons) and *p *< 0.005 (10 comparisons) for the quantified and white matter normalized analyses, respectively.

The Bland-Altman test [19] was used to assess the agreement between corresponding grey matter measurements. The mean difference, standard deviations, and the 95% limits of agreement were calculated and plotted. The rCBF measures were plotted against each other for each subject individually using linear regression analyses; the linear slopes were determined; and the coefficient of determination [*r*^2^] between the two methods was calculated.

## Results

### Blood gas analyses

Air bubbles in one syringe and malfunction of one arterial catheter left a total of 11 subjects for this analysis (Table 1). There was no significant change in blood gas levels and haemoglobin concentration between the first and the second PET scan. However, when comparing the averaged blood gas levels of the two PET scans to the gas values measured immediately after CT, there was a modest, but significant drop in P_a_CO_2 _of approximately 0.3 kPa or 2 mmHg. At the same time, there was a slight, but non-significant (*p *= 0.07), increase in P_a_O_2 _of approximately 1.0 kPa or 7 mmHg. The data indicate slight hyperventilation during the performance of perfusion CT and confirm that no discernable effects of the approximately 100-mL blood drawn during PET scanning could be found in the haemoglobin concentration.

**Table 1:** Blood gas measurements

	RCBF PET	RCBF CT	
(*n *= 11)	Mean ± SD	Mean ± SD	*p *Value
P_a_CO_2 _(kPa)	5.51 ± 0.50	5.23 ± 0.42	< 0.01
P_a_O_2 _(kPa)	13.80 ± 1.37	14.77 ± 1.19	NS
ctHb (mmol/L)	8.22 ± 0.69	8.12 ± 0.73	NS
sO_2 _(%)	0.98 ± 0.01	0.99 ± 0.00	NS

RCBF, regional cerebral blood flow; CT, computed tomography; PET, positron-emission tomography; SD, standard deviation; NS, non-significant; P_a_CO_2_, arterial tension of carbon dioxide; P_a_O_2_, arterial tension of oxygen; ctHb, haemoglobin concentration; sO_2_, oxygen saturation.

### RCBF measures

**Figure 2:**
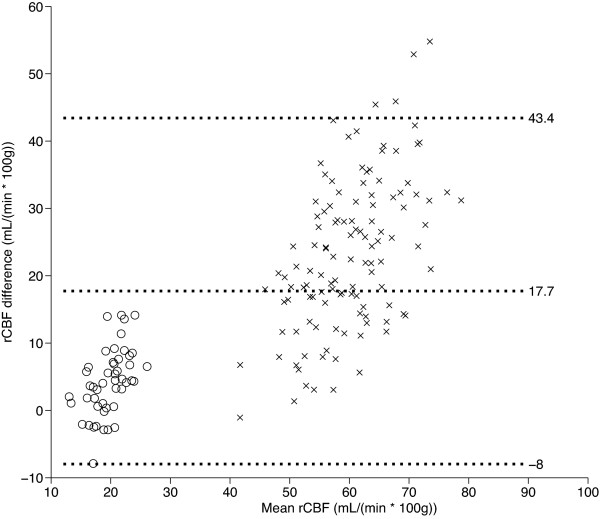
**Bland-Altman plot of the difference between rCBF CT and rCBF PET against their mean values**. The middle line indicates the mean difference. The outer lines indicate 95% limits of agreement. The rCBF CT values are biased and are all larger than the rCBF PET values, and the difference increases with increasing mean rCBF values. Cross mark, grey matter; empty circle, white matter.

There was no significant difference between the two rCBF PET measurements. The absolute measurements are summarised in Table 2. The average volume-weighted rCBF PET measurements were 17.4 ± 2.0 mL min^-1 ^100 g^-1 ^(mean ± standard deviation) for the white matter and 48.7 ± 5.0 mL min^-1 ^100 g^-1 ^for the grey matter with a between-subject regional coefficient of variance [COV] in the 10% to 17% range. In all regions but one white matter ROI, the absolute rCBF measurements with perfusion CT were significantly higher than the PET measurements even after a rigid Bonferroni correction. The average volume-weighted rCBF measurements with perfusion CT were 21.8 ± 3.4 mL min^-1 ^100 g^-1 ^for the white matter and 71.8 ± 8.0 mL min^-1 ^100 g^-1 ^for the grey matter. The COVs were higher than the rCBF PET measurements in 11 of 14 ROIs ranging from 11% to 28%. The mean increase compared to rCBF PET was 4.4 ± 3.3 mL min^-1 ^100 g^-1 ^in the white matter and 23.1 ± 8.5 mL min^-1 ^100 g^-1 ^in the grey matter (Table 2). The lower and upper 95% limits of the grey and white matter changes from PET to CT rCBF were -8.0 and 43.4 mL min^-1 ^100 g^-1^, respectively (Figure 2). The overall mean volume-weighted grey matter/white matter ratio was 2.78 ± 0.25 for rCBF PET, and it was significantly higher for perfusion CT, 3.34 ± 0.48 (Table 3). On the regional level, the ratios were significantly higher in the right caudate nucleus, in the frontal and parietal cortices, and in the left parietal cortex. Interestingly, compared to the absolute measurements, the regional COVs were reduced in seven out of ten regions for rCBF PET, but increased in eight out of ten for rCBF CT. Thus, normalization to the white matter reduced the volume-weighted grey matter COV for rCBF PET from 10.4% to 9.1%, while it was increased for rCBF CT from 11.2% to 14.3%.

**Table 2:** RCBF measurements from PET and perfusion CT scanning

		PET	COV	CT	COV	ΔCBF	
Side	ROI	Mean ± SD	%	Mean ± SD	%	Mean ± SD	*p *Value
Right	Caudate nucleus	48.7 ± 6.3	12.9	71.6 ± 10.1	14.2	22.9 ± 12.2	< 0.05
	Putamen	50.5 ± 7.1	14	73.9 ± 13.9	18.7	21.5 ± 13.9	< 0.05
	Frontal cortex	49.4 ± 6.8	13.7	74.4 ± 9.1	12.3	25.0 ± 9.3	< 0.05
	Parietal cortex	45.0 ± 6.7	14.9	72.0 ± 8.5	11.8	27.1 ± 10.7	< 0.05
	Occipital cortex	45.1 ± 5.7	12.7	68.5 ± 15.5	22.7	23.4 ± 15.3	< 0.05
	Anterior white matter	17.6 ± 3.0	17.1	18.9 ± 5.2	27.7	1.3 ± 5.9	NS
	Posterior white matter	18.0 ± 2.5	14.1	22.5 ± 4.2	18.7	4.5 ± 3.7	< 0.05
Left	Caudate nucleus	49.7 ± 4.9	9.9	69.6 ± 10.7	15.4	19.9 ± 10.6	< 0.05
	Putamen	51.3 ± 5.1	10	73.0 ± 11.9	16.4	19.9 ± 11.8	< 0.05
	Frontal cortex	51.5 ± 6.3	12.3	73.3 ± 9.2	12.6	21.8 ± 8.7	< 0.05
	Parietal cortex	46.8 ± 5.5	11.8	73.3 ± 8.3	11.4	26.5 ± 8.7	< 0.05
	Occipital cortex	46.4 ± 7.2	15.6	67.1 ± 15.9	23.7	20.6 ± 14.0	< 0.05
	Anterior white matter	18.4 ± 2.9	15.5	23.2 ± 3.9	17	4.8 ± 4.8	< 0.05
	Posterior white matter	16.5.± 2.0	12	22.7 ± 4.4	19.4	6.2 ± 4.3	< 0.05
	Volume weighted white matter	17.4 ± 2.0	11.7	21.8 ± 3.4	15.8	4.4 ± 3.3	< 0.05
	Volume weighted grey matter	48.7 ± 5.0	10.4	71.8 ± 8.0	11.2	23.1 ± 8.5	< 0.05

CBF, cerebral blood flow; PET, positron-emission tomography; CT, computed tomography; ROI, region of interest; SD, standard deviation; COV, coefficient of variance; NS, non-significant. *N *= 12. RCBF units are in millilitre per minute per 100 g. The *p *values are Bonferroni-corrected.

**Table 3:** Relative rCBF grey matter measurements from PET and perfusion CT scanning normalized to white matter

		PET	COV	CT	COV	ΔCBF	
Side	ROI	Mean ± SD	%	Mean ± SD	%	Mean ± SD	*p *Value
Right	Caudate nucleus	2.77 ± 0.26	9.4	3.32 ± 0.55	16.6	0.56 ± 0.40	< 0.05
	Putamen	2.90 ± 0.30	10.4	3.39 ± 0.62	18.3	0.44 ± 0.65	NS
	
	Frontal cortex	2.81 ± 0.30	10.5	3.47 ± 0.60	17.2	0.66 ± 0.52	< 0.05
	Parietal cortex	2.56 ± 0.35	13.6	3.37 ± 0.67	19.8	0.81 ± 0.67	< 0.05
	Occipital cortex	2.58 ± 0.34	13.3	3.17 ± 0.64	20.2	0.59 ± 0.72	NS

Left	Caudate nucleus	2.84 ± 0.33	11.6	3.24 ± 0.62	19	0.40 ± 0.45	NS
	Putamen	2.96 ± 0.28	9.4	3.36 ± 0.66	19.7	0.37 ± 0.66	NS
	
	Frontal cortex	2.94 ± 0.31	10.4	3.41 ± 0.56	16.3	0.48 ± 0.52	NS
	Parietal cortex	2.67 ± 0.34	12.7	3.43 ± 0.64	18.5	0.76 ± 0.70	< 0.05
	Occipital cortex	2.65 ± 0.40	15	3.12 ± 0.77	24.8	0.47 ± 0.69	NS
	
	Volume weighted grey matter	2.78 ± 0.25	9.1	3.34 ± 0.48	14.3	0.56 ± 0.47	< 0.05

CBF, cerebral blood flow; PET, positron-emission tomography; CT, computed tomography; ROI, region of interest; SD, standard deviation; COV, coefficient of variance; NS, non-significant. *N *= 12. The *p *values are Bonferroni-corrected.

**Figure 3:**
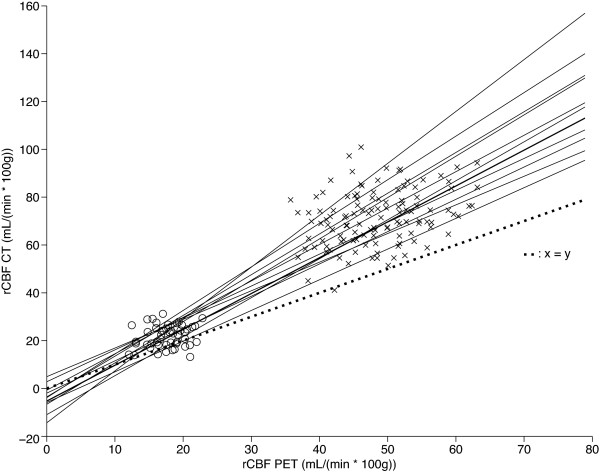
**Scatter plot of rCBF CT against rCBF PET**. Linear regression lines are shown for each subject (*n *= 12). The line of perfect agreement is indicated. The rCBF CT values are clearly biased towards higher rCBF values, and the regression slopes are all above 1.0. Cross mark, grey matter; empty circle, white matter.

The Bland-Altman plots demonstrated a bias for rCBF CT that increases from lower to higher mean grey and white matter values (Figure 2). The linear slopes between the two methods when including both white and grey matter values were significantly different from zero in all individuals with an average *r*^2 ^of 0.89 (range 0.82 to 0.96) and an average slope of 1.56 (range 1.20 to 2.17; Figure 3). When only the grey matter regions were analysed, the slopes were only significant in 4 of 12 subjects. These four subjects had *r*^2 ^of 0.40 to 0.50 and slopes between 0.5 and 1.75.

## Discussion

In this study, we have compared the quantitative rCBF values that can be obtained by two imaging techniques,^15^O-H_2_O PET and perfusion CT. To date, studies that directly validate perfusion CT in healthy subjects have been very scarce. The majority of studies concern patients with cerebrovascular disease, and validation has been against either the stable xenon-CT method [20,21] or^15^O-H_2_O PET [22]. Although rCBF can be derived from the non-ischemic hemisphere in stroke patients, the design is suboptimal. It is quite possible that regional perfusion in the undamaged hemisphere is influenced to some degree by either a subclinical tissue pathology, a generalised micro- or macrovascular disease, remote functional effects of neural damage (diaschisis), or a co-morbidity (cardiac function, pulmonary disease). Particularly the quality of the bolus input gives errors in the rCBF determination, e.g. bolus buffering in the lungs. Therefore, a reduced cardiac output will systematically decrease rCBF [23]. Similarly, in patients with carotid occlusion, selection of a single arterial input function will cause increased delay and dispersion of the contrast agent to the ischemic areas and thus, underestimate rCBF by 15% to 20% [24].

As the greater part of the validation studies is aimed at cerebrovascular diseases, the regions used focus on the major cerebral artery territories or whole hemispheres. Thus, the rCBF measures are a heterogeneous mixture derived from both the white and grey matter tissues, vascular volumes, and cerebrospinal fluid spaces, but the relative weights of the individual tissue components are unknown [21,22,24-26]. This will render direct comparison between our quantitative grey matter rCBF CT values and these studies difficult.

In studies where the white and grey matter rCBF CT values are available, these range from 14 to 30 mL min^-1 ^100 g^-1 ^and 40 to 70 mL min^-1 ^100 g^-1^, respectively, with a COV of 25% to 30% [6,20,23,27]. Although mostly derived from patient studies, these results are quite similar to the results we found in normal healthy subjects. We found average volume-weighted rCBF CT measurements of 21.8 ± 3.4 mL min^-1 ^100 g^-1 ^for the white matter and 71.8 ± 8.0 mL min^-1 ^100 g^-1 ^for the grey matter. The relative regional between-subject COV ranged from 11% to 28%. The rCBF CT values were significantly larger than the average volume-weighted rCBF PET measurements by 25% in the white matter and 47% in the grey matter. The absolute values were 17.4 ± 2.0 mL min^-1 ^100 g^-1 ^for the white matter and 48.7 ± 5.0 mL min^-1 ^100 g^-1 ^for the grey matter with a COV in the 10% to 17% range. Our findings correspond to the values previously reported in the literature using this technique [7,8,13,28,29]. In a larger Japanese study encompassing 70 healthy subjects spanning 11 institutions, the overall average rCBF for cerebral cortical regions were 42.7 ± 6.3 mL min^-1 ^100 g^-1 ^with a COV of 14.6% [30].

When normalizing to the white matter, the relative regional grey matter COV was nominally lower with PET compared to CT in 10 of 14 ROIs. One explanation for the lower COV with PET was that we used the average of two measurements. This was not done for perfusion CT to keep the radiation dose within acceptable limits. To our knowledge, a test-retest study of baseline rCBF CT has not been reported on healthy subjects.

In the study design, we have tried to limit the variation between the two techniques further by performing same-day measurements within 1 to 2 h. A further source of variation, which has not been considered in previous validation studies, is the impact that changes of the pre-scan arterial blood gas status might have on rCBF [29]. The P_a_CO_2 _decreased significantly by 2 mmHg from PET to CT scanning, suggesting slight hyperventilation. Hyperventilation decreases rCBF by washing out P_a_CO_2 _by approximately 2% per millimetre of mercury [31,32]. This would decrease the rCBF CT measurements by 4% and further increase the difference between techniques if corrected for. The hyperventilation itself may have been caused by anticipatory anxiety in the CT scanning session possibly associated to the procedure itself. Mood states in scanning sessions have been investigated by Matthew et al. [28], and a trend was found for anticipatory anxiety to be lowered from the first to second scans. This may have been forestalled by letting the subjects rest in the CT scanner for several minutes before scans and maybe even perform a 'sham-scan' before scans.

The bias between techniques was not constant, but increased with the increasing rCBF value (Figure 2), which indicates a logarithmic influence. It is possible that there are global effects in a measurement that influences the regional values. This could be between-subject differences, physiological fluctuations, or methodological errors pertaining to the measurement of the input function and the involved corrections [32,33] or in the selection of the venous ROI and the arterial input function in perfusion CT [23,34]. One strategy is to normalize the rCBF to the tissue that systematically co-varies with the global fluctuations and is not affected by isolated pathological processes. We examined this hypothesis using the white matter as a reference tissue. For the overall grey matter, the normalized rCBF in perfusion CT was only 20% larger than that for PET. Thus, more than half of the difference between techniques can be explained by global fluctuations affecting both tissues alike, but there is a residual effect manifest as a larger contrast between the white and grey matter tissues in perfusion CT. Interestingly, normalization to the white matter reduced the grey matter COV in PET, but increased the COV in perfusion CT, indicating that there are individual grey/white matter differences to be considered. Thus, prior to the use of tissue normalization in a clinical setting, it is essential that effects of bias and noise are well understood.

One aspect that also needs to be examined is the effect of differences in resolution, the partial volume effect [PVE]. The PVE will of course affect not only PET values, but also PCT values, as the resolution of both methods is insufficient to accurately quantify the rCBF in the cortical grey matter. The object of the paper, however, was not so much to measure 'true' cortical rCBF, but to compare two methods under clinical conditions. So the strategy was to have the PVE affect the two methods to the same degree, rather than to introduce a new level of complexity and potential bias by PVE correction through e.g. tissue-segmented MRI. This was done by securing comparable image resolutions and an accurate image registration between the two imaging modalities. Thus, any error caused by tissue heterogeneity in a given ROI would affect the sampled values to the same degree. We do not believe that the differences between methods can be related to image resolution.

The two methods,^15^O-H_2_O-PET and perfusion CT, are inherently different since perfusion CT relies on the dynamic behaviour of a non-diffusible intravascular iodine medium, whereas^15^O-H_2_O-PET relies on a tracer that is freely diffusible into the tissues. Strictly speaking, the term 'rCBF' should only be reserved to denote the volume flow rate of blood though a functional tissue that has the ability to exchange nutrients and waste products, thus, the capillary blood flow. However, a purely intravascular tracer, as iodine contrast, will distribute to all vascular segments. Thus, a fundamental flaw with perfusion CT is the presence of high-contrast signals in regions without a functional tissue and a capillary bed such as the choroid plexus, arteries, arterioles, venules, veins, and sinuses. An example can be seen in Figure 1, where a draining vein in the left occipital region has an rCBF signal increase on perfusion CT without any discernable signal on^15^O-H_2_O-PET, indicating the absence of a functional tissue. The most common strategy is to eliminate vascular pixels in the CT images before calculation of rCBF. A simple regional rCBV threshold of 8 mL/100 g has been suggested as the most accurate [6]. This threshold, however, was not feasible in our study as large and irregular sections of the brain parenchyma were excluded from analyses. We, thus, chose a threshold of 14.4 mL/100 g that respected tissue integrity and kept the rCBF CT images legible for clinical use. The grey matter rCBV has been measured to 3 to 4 mL/100 g [30,35], so both thresholds are somewhat above the normal tissue rCBV level. In the ROI definitions, we carefully omitted obvious larger vascular structures, but there is definitely a contribution from smaller non-capillary vessels and probably also from a PVE from larger vessels. We regard that the blood volume influenced the signal as the dominant error source in the overestimation of rCBF CT values, in the increased contrast between the white and grey matter, and as an important regional noise contribution. This has been recognized previously as well [6,36,37].

Although biased, we found that the rCBF CT does correlate with rCBF PET for each individual over a broad range of values from the white to the grey matter (Figure 3), but poorly if only grey matter rCBF values were considered. Previous studies in patients have found *r*^2 ^ranging from 0.5 to 0.8 with significant linear regression slopes of 0.7 to 1.4 [20,22,38,39] and in healthy subjects, *r*^2 ^from 0.4 to 0.9 with slopes 1.0 to 1.55 [6,25]. The significant correlations signify that rCBF CT does deliver a perfusion-weighted signal, but with a tendency to overestimate the values particularly for highly perfused regions.

## Conclusion

Although perfusion CT is an attractive, widely available, relatively cheap, rapid, and easily performed method, we have not been able to confirm some of the previously published reports of high accuracy performed mainly on patients with cerebrovascular disease. Perfusion CT is not accurate enough in the current setting. In healthy subjects, perfusion CT does deliver a perfusion-weighted signal, but with a tendency to overestimate the values particularly for highly perfused regions. The average overestimation of rCBF in the grey matter of 47% is unacceptably high. Neither with respect to absolute quantification nor perfusion distribution can rCBF CT measures substitute rCBF PET, primarily because of the confounding effects of blood volume. This does not exclude a useful role in patient management, which must, however, be evaluated in separate investigations with the normative data in mind.

## Abbreviations

CBF: cerebral blood flow; CT: computed tomography;^15^O-H_2_O:^15^O-labelled water; PET: positron-emission tomography; rCBF: regional CBF.

## Competing interests

The authors declare that they have no competing interests.

## Authors' contributions

IL conceived the study. JG, LH, and IL participated in the design of the study. JG and IL coordinated the study, and JG carried out the scannings. RP performed the statistical analysis. All authors drafted the manuscript and read and approved the final manuscript.
